# Effects of the Electric Double Layer Characteristic and Electroosmotic Regulation on the Tribological Performance of Water-Based Cutting Fluids

**DOI:** 10.3390/mi14112029

**Published:** 2023-10-31

**Authors:** Ruochong Zhang, Wenshuai Liu, Zhiqiang Luan, Yu Xia, Ying Wang, Xiaodong Hu, Faisal Z. Duraihem, Xuefeng Xu

**Affiliations:** 1 College of Mechanical Engineering, Zhejiang University of Technology, Hangzhou 310023, China; zhangruochong@zjut.edu.cn (R.Z.); 2112102109@zjut.edu.cn (W.L.); 1112002019@zjut.edu.cn (Z.L.); 1112102004@zjut.edu.cn (Y.X.); 2112002059@zjut.edu.cn (Y.W.); hooxoodoo@zjut.edu.cn (X.H.); 2 Key Laboratory of Special Purpose Equipment and Advanced Processing Technology, Ministry of Education and Zhejiang Province, Zhejiang University of Technology, Hangzhou 310023, China; 3 Department of Mathematics, College of Science, King Saud University, P.O. Box 2455, Riyadh 11451, Saudi Arabia; faldureiham@ksu.edu.sa

**Keywords:** electroosmosis, electrical double layer, zeta potential, water-based cutting fluid, tribological performance

## Abstract

The electroosmosis effect is a complement to the theory of the traditional capillary penetration of cutting fluid. In this study, based on the electric double layer (EDL) characteristics at friction material/solution interfaces, the influences of additives and their concentrations on capillary electroosmosis were investigated, and a water-based cutting-fluid formulation with consideration to the electroosmosis effect was developed. The lubrication performance levels of cutting fluids were investigated by a four-ball tribometer. The results show that the EDL is compressed with increasing ionic concentration, which suppresses the electroosmotic flow (EOF). The specific adsorption of OH^−^ ions or the dissociation of surface groups is promoted as pH rises, increasing the absolute zeta potential and EOF. The polyethylene glycol (PEG) additive adsorbed to the friction material surface can keep the shear plane away from the solid surface, reducing the absolute zeta potential and EOF. The electroosmotic performance of cutting fluid can be improved by compounding additives with different electroosmotic performance functions. Furthermore, electroosmotic regulators can adjust the zeta potential by the electrostatic adsorption mechanism, affecting the penetration performance of cutting fluid in the capillary zone at the friction interface. The improvement in the tribological performance of cutting fluid developed with consideration given to the electroosmosis effect is attributed to the enhancement of the penetration ability of the cutting fluid and the formation of more abundant amounts of lubricating film at the interface.

## 1. Introduction

Cutting fluids play an important role in machining by providing lubrication, cooling, cleaning, and rust prevention to improve the surface quality of workpiece and extend tool life [1]. Research conducted by Chen et al. [2] and Zhang et al. [3] demonstrated that the micro-textures on the tool’s surface could promote the penetration of cutting fluid into the tool–chip friction interface to improve lubrication and reduce cutting force. Diniz et al. [4] and Liu et al. [5] stated that high-pressure coolant technology was conducive to the penetration of cutting fluid into the tool–workpiece friction interface, effectively providing cooling and lubrication effects. The penetration ability of cutting fluid at the tool–chip/workpiece friction interface directly affects the surface formation process and machining quality, thus attracting more and more research attention to the area. Godlevski et al. [6,7] pointed out that capillary penetration was the predominant factor to consider in studying cutting fluid’s ability to enter the cutting zone and proposed three stages for cutting-fluid penetration into the friction interface capillary: liquid phase entry, micro-droplet explosion, and filling of the capillary by gas phase. The kinetic theory of the traditional capillary penetration of cutting fluid analyzed the comprehensive effects of atmospheric pressure, capillary-wall viscous resistance, and capillary driving force [8,9]. Nakayama et al. [10,11,12,13] demonstrated that triboplasma could be generated in the gap of the friction interface regardless of dry friction or boundary lubrication, which could further generate a self-excited electric field at the friction interface. Feng et al. [14] established that a self-excited electric field of 150–1090 V/cm could be generated in the capillary at the tool–chip friction interface, with the electric field direction pointing to the inner end of the capillary; this is comparable to the self-excited electric field in the capillary at the steel–ceramic friction interface (300–600 V/cm) found by Luan et al. [15] through numerical simulation. In addition, the two research also pointed out that the self-excited electric field could induce the capillary electroosmotic behavior of cutting fluid. Therefore, enhancing the capillary electroosmotic performance of cutting fluid to promote penetration at the friction interface has practical significance for the full utilization of the lubrication performance aspects of cutting fluid.

Carbide, high-speed steel, and ceramic tools are widely used in machining iron-based materials. The cutting fluid exhibits different zeta potentials on the surface of these friction materials (steel or ceramic), thus affecting its electroosmotic performance in the capillary zone at the friction interface. Feng et al. [14] investigated the zeta-potential characteristics of deionized water on the surfaces of tools or workpieces. They discovered that cemented carbide tools, mild steel, stainless steel, polyethylene, and other workpieces exhibited negative zeta potential in deionized water. Additionally, an electroosmotic promoter, such as sodium lauriminodipropionate (SLI), could increase the absolute value of the zeta potential, promoting the EOF of deionized water in the capillary zone at the friction interface and thus reducing cutting forces. Xu et al. [16] demonstrated that the addition of 0.2 mmol/L of electroosmotic promoter (such as 3-[(3-cholamidopropyl)-dimethylammonio]-1-propanesulfonate (CHAPS)) could enhance the electroosmotic performance levels of SiO_2_ nanofluid lubricant at the steel–steel and steel–ceramic friction interfaces and improve the tribological performance of the lubricant. Generally, the zeta-potential characteristics (amplitude and polarity) depend on the physical and chemical properties between solid surface and liquid. Falahati et al. [17] demonstrated the effect of ionic strength and pH value on the zeta potential at the PMMA/NaCl interface. The results showed that the absolute value of the zeta potential decreased linearly with an increase of the logarithmic value of ionic strength and increased with a rising pH value (in the alkaline range). Zimmermann et al. [18] investigated the dependence of zeta potential on pH in 1 mmol/L KCl solution on three polymer film surfaces. They observed that in the alkaline pH range, the zeta potential was negative, and the absolute value increased with the rise of the pH value in the background electrolyte solution. Zhao et al. [19] and Brown et al. [20] also indicated that the absolute value of the zeta potential decreased with increased ionic strength, which was due to the compression of the EDL by high ionic strength to reduce the charge density in the EDL. Additionally, ionic surfactants adsorbed to charged surfaces by electrostatic or van der Waals forces could significantly affect the zeta potential and have been commonly used to regulate EOF [21,22,23,24,25,26,27]. At present, there is still little research on zeta potentials at friction material/cutting-fluid interfaces, and the influences of cutting-fluid components on zeta potential and EOF need to be further revealed.

Water-based cutting fluids have developed rapidly because of their low cost, excellent cooling performance, and environmental friendliness [28,29,30,31,32]. Water-based cutting fluids used in actual production usually include lubricity additives, extreme pressure (EP) additives, pH buffers, rust inhibitors, biocides, and other components [33]. Water-soluble polyethers and synthetic esters have frequently been utilized in water-based cutting fluid due to their excellent lubrication performance levels [34,35,36]. Gu et al. [37] demonstrated that triethanolamine borate and polyether additive showed a good synergistic effect on tribological performance. Goindi et al. [38] discovered that polyethylene glycol (PEG) as a lubricity additive could improve machining surface quality. Benedicto et al. [39] indicated that the addition of synthetic ester additives to water-based cutting fluid could significantly improve tribological performance and extend tool life. In the context of high temperature, heavy load, or boundary lubrication, EP additives play an important role in improving the load-bearing capacity of lubrication film. An et al. [40] found that nonylphenol polyoxyethylene ether phosphate ester could improve the extreme pressure anti-wear performance of water-based cutting fluid and that it increased the maximum non-seizure load from 88 N to 212 N when 0.3% was added. Zhao et al. [41] used new organic molybdenum and organic boron as EP additives for water-based cutting fluid. They observed that the two had a synergistic effect, and the extreme-pressure lubrication coefficient of the cutting fluid increased significantly after using the composite EP additive. Yang et al. [42] claimed that water-soluble phosphate ester as an additive for water-based cutting fluid could effectively improve the lubrication performance at a TC4-YG8 friction interface. In addition, the pH value of water-based cutting fluid is related to its anti-rust performance and anti-corrosion performance. The addition of a pH buffer can maintain the pH of the cutting-fluid system within a reasonable range. Commonly used pH buffers include triethanolamine (TEA), borax, etc. [43,44,45]. The EDL characteristics of various additive solutions at the friction material surface are more complex. Therefore, clarifying the zeta-potential characteristics of the friction material/solution interface has an important supporting effect in the design of cutting-fluid formulation based on the electroosmosis effect.

In this paper, the EOF velocities of solutions of water-based cutting-fluid additives and their zeta-potential characteristics on the surfaces of steel and ceramic were studied, and the varying influences of additives on the electroosmotic performance levels of cutting fluid were revealed. A cutting-fluid formulation that considered the electroosmosis effect was obtained based on the study of the EOF and zeta-potential characteristics of compound cutting fluids with preferred additives. The effects of electroosmotic regulators 3-[(3-cholamidopropyl)-dimethylammonio]-1-propanesulfonate (CHAPS, electroosmotic promoter) and cetyltrimethylammonium bromide (CTAB, electroosmotic suppressant) on the tribological performance levels of cutting fluid at the steel–steel interface were investigated by four-ball tribometer. The penetration ability of cutting fluid was evaluated by elemental analysis of worn surfaces.

## 2. Experimental Details

### 2.1. Preparation of Water-Based Cutting Fluids

Lubricity additive, EP additive, and pH buffer were selected to form the basic formula of a water-based cutting fluid. Additionally, CHAPS and CTAB were selected as electroosmotic regulators to further optimize the electroosmotic performance of the basic formula. Information on the chemical reagents used is listed in Table 1. To investigate the electroosmotic performance levels of water-based cutting-fluid additives, certain amounts of additives were ultrasonically dispersed into deionized water to prepare the following solutions: polyethylene glycol (PEG) lubricity additive solutions (1 wt%, 3 wt%, 5 wt%, 10 wt%, and 15 wt%), potassium tetraborate (B_4_K_2_O_7_·4H_2_O) or fatty alcohol polyoxyethylene ether phosphate (MOA-3P and MOA-9P; the difference between the two is the ratio of RO(CH_2_CH_2_O)_n_PO(OH)_2_ and [RO(CH_2_CH_2_O)_n_]_2_PO(OH)) EP additive solutions (0.05 wt%, 0.15 wt%, 0.25 wt%, 0.5 wt%, 0.75 wt%, 1 wt%, 3 wt%, and 5 wt%), and triethanolamine (TEA) pH buffer solutions (2.55 × 10^−7^ wt%, 2.55 × 10^−5^ wt%, 2.55 × 10^−3^ wt%, 3 wt%, 5 wt%, 10 wt%, and 15 wt%). The determination of additive concentration levels was based on the common proportions of additives in water-based cutting fluids [46]. According to the results of the fluids’ electroosmotic performance, a water-based cutting fluid with excellent electroosmotic performance was designed and prepared (and named WCF). Then, CHAPS and CTAB with concentrations of 0.05, 0.2, 0.4, and 0.8 mmol/L were added to study their effects on the electroosmotic performance and tribological performance of the cutting fluid.

### 2.2. Capillary Electroosmotic Experiment

**Figure 1:**
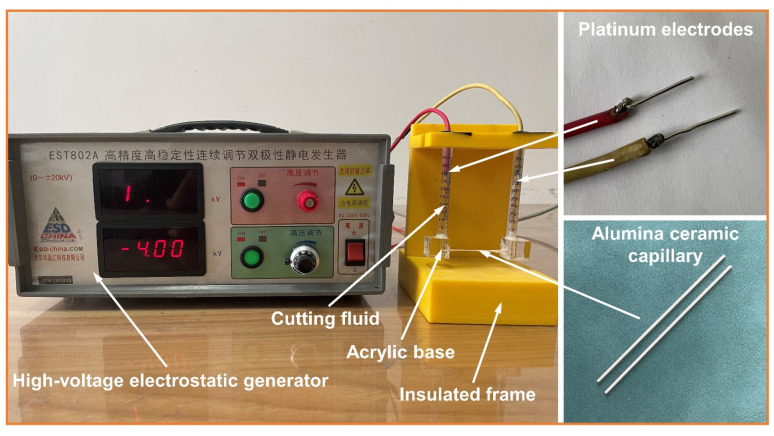
Photograph of capillary EOF velocity measurement device of cutting fluid.

Figure 1 shows the capillary EOF-velocity measurement device for cutting fluid. Due to the difficulty of purchasing capillaries made of AISI 52100 steel on the market, alumina ceramic capillaries with an inner diameter of 300 μm and a length of 50 mm were selected for testing; their EDL characteristics were similar to those of AISI 52100 steel [15]. Both ends of the capillary were connected to a syringe by an acrylic base. The cutting fluid was injected into the syringe from one side to avoid air bubbles. The axial electric field in the capillary was applied by an EST802A high-voltage electrostatic generator (Huajinghui Technology Co., Ltd., Beijing, China). The syringes on both sides were connected to the negative high-voltage electrode and grounding electrode, respectively. In the test, the output voltage of the electrostatic generator was −4 kV, which induced an axial electric field in the capillary with an intensity of about 800 V/cm. The time and the movement direction were recorded when 20 μL cutting fluid moved through the capillary, and then the EOF velocity was calculated. Each test was repeated at least three times at room temperature, and the average value for EOF velocity was recorded.

### 2.3. Streaming Potential Testing and Zeta-Potential Calculation

**Figure 2:**
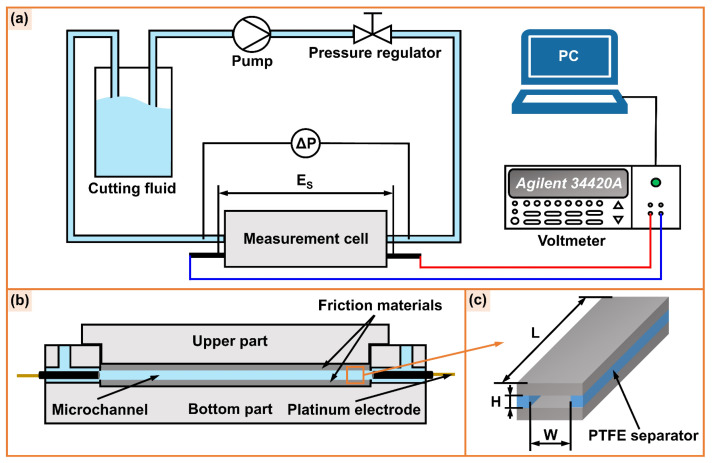
Schematic diagram of (**a**) the tangential streaming potential test system, (**b**) the measurement cell, and (**c**) the parallel-plate microchannel.

The tangential streaming potential measurement technique is widely used to determine the zeta potential at the solid/liquid interface because the calculated zeta potential more accurately reflects the EDL characteristics at the interface [47,48]. Figure 2a shows the schematic diagram of the tangential streaming potential method. The friction materials (AISI 52100 steel or alumina ceramic) were seated in the measurement cell (Figure 2b) and separated by polytetrafluoroethylene (PTFE) separators to form a microchannel (Figure 2c). During the test, the cutting fluid, driven by a pump, circularly flowed through the measurement cell. Additionally, the pressure difference ∆*P* on both sides of the microchannel was adjusted by a pressure regulator, which increased linearly from 0.02 MPa to 0.1 MPa. The potential difference between the two sides of the microchannel (streaming potential *E_S_*) was measured by an Agilent 34420A voltmeter (Agilent Technologies Inc., Shanghai, China). According to the streaming potential coefficient (*E_S_*/∆*P*), the zeta potential at the friction material/solution interface was calculated as follows:

(1) $$\zeta =\frac{E_{S}}{\Delta P}\cdot \frac{\eta }{\varepsilon }\cdot \lambda$$

where *ζ* is the zeta potential, *η* is the solution viscosity, *ε* = *ε*_0_*ε_r_* is the solution permittivity, and *λ* is the solution bulk conductivity.

Due to the excellent electrical conductivity of AISI 52100 steel, numerous ions flow back through the material bulk when measuring the streaming potential at the AISI 52100 steel/solution interface [49]. Therefore, the total conductance (including solid bulk conductance, surface conductance, and solution conductance) was used in this study to calculate the zeta potential at the AISI 52100 steel/solution interface [50]:

(2) $$\zeta =\frac{E_{S}}{\Delta P}\cdot \frac{\eta }{\varepsilon }\cdot \frac{L}{\mathrm{WH}}\cdot G_{t}$$

where *L*, *W*, and *H* are the length, width, and height of the microchannel, respectively, corresponding to a size of 160 × 10 × 0.3 mm; and *G_t_* is the total conductance of the AISI 52100 steel microchannel filled with solution; and *G_t_* = 1/*R_t_*. *R_t_* is the total resistance of the AISI 52100 steel microchannel filled with solution, as measured by the Keithley 6517B electrometer.

In addition, the absolute value of the zeta potential at the alumina ceramic/solution interface would be underestimated by Equation (1) in the case of a solution with low ion concentration. Therefore, the *EDL* conductivity was used in this study to calculate the zeta potential at the alumina ceramic/solution interface [15]:

(3) $$\zeta =\frac{E_{S}}{\Delta P}\cdot \frac{\eta }{\varepsilon }\cdot \lambda_{\mathrm{EDL}}$$

where *λ_EDL_* is the *EDL* conductivity of the alumina ceramic microchannel filled with solution, and the galvanostatic four-electrodes system combined with electrochemical impedance spectroscopy was used for measurement and calculation.

### 2.4. Tribological Tests

The MMW-1 multi-specimen test system (Jinan PuYe Mechanic & Electronics Technology Co., Ltd., Jinan, China) was used to conduct the four-ball friction test under full immersion lubrication. The specific test conditions are listed in Table 2. The selection of load and rotational speed was based on industry-standard NB/SH/T 0189-2017 (Standard test method for wear preventive characteristics of lubricating fluid−four-ball method). Before the test, the steel balls and ball pot were ultrasonically cleaned in acetone. Each test was repeated at least three times at room temperature, with the coefficient of friction (COF) being recorded and averaged. After each test, the VW-6000 microscope system (Keyence Co., Ltd., Osaka, Japan) was employed to measure the wear scar diameter (WSD) of the steel ball. The worn surface morphologies and element components of steel balls were examined using a Sigma HV-01-43 scanning electron microscope (SEM) (Zeiss Co., Ltd., Oberkochen, Germany) equipped with an energy-dispersive spectrometer (EDS).

## 3. Results and Discussion

### 3.1. Electroosmotic Performance Levels of Single-Component Additive Aqueous Solutions

#### 3.1.1. EOF Velocity

**Figure 3:**
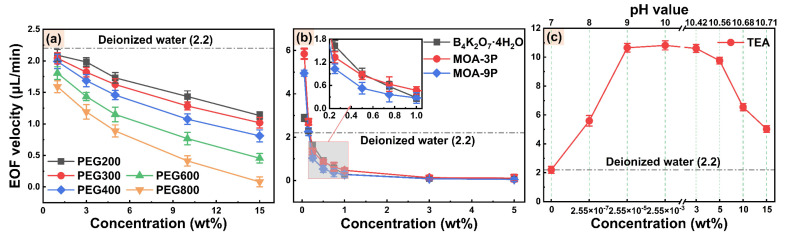
EOF velocities of (**a**) lubricity additive, (**b**) EP additive, and (**c**) pH buffer aqueous solutions.

Figure 3 shows the EOF velocities of lubricity additive, EP additive, and pH buffer additive aqueous solutions. It can be observed from Figure 3a that the EOF velocities of PEG solutions (molecular weights from 200 to 800) in the concentration range from 1 wt% to 15 wt% are lower than that of deionized water and decrease with increased concentrations and molecular weights. This is probably because the dynamic viscosity of PEG solution increases with increasing molecular weight and concentration (Figure S1a), resulting in increased capillary-wall viscous resistance and thus suppressed EOF [51]. Additionally, it can be observed from Figure 3b that the EOF velocities of three EP additive solutions decrease rapidly with increasing concentration, and the EOF velocity of MOA-3P solution is slightly higher than that of MOA-9P and the potassium tetraborate solution. Additionally, within the concentration range of 0.15 wt%, the EOF velocities of the three EP additive solutions are higher than that of deionized water. However, when the concentration exceeds 1 wt%, the EOF velocities of the three EP additive solutions are almost 0. This may be related to the significant boost in the dynamic viscosity (except for the potassium tetraborate) and bulk conductivity of all three EP additive solutions above 1 wt%, as shown in Figure S1b and Figure S2b, respectively. When the conductivity is too high, the joule heating effect causes bubbles in the capillary solution, suppressing or even interrupting the EOF [52]. Additionally, the EOF velocity of TEA solution exhibits a trend of first increasing, then leveling off, and finally decreasing, within a pH range from 7 to 10.71, as presented in Figure 3c. The EOF velocity of TEA solution is higher than that of deionized water in all test concentrations, although its dynamic viscosity (Figure S1c) and bulk conductivity (Figure S2c) increased with increased concentration. This might be because the capillary-wall surface charge is affected by the pH value, which alters the zeta potential and further affects the EOF velocity [53].

#### 3.1.2. Streaming Potential and Zeta Potential

**Figure 4:**
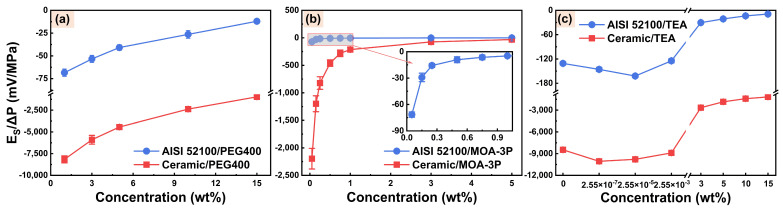
Streaming potentials of (**a**) PEG400, (**b**) MOA-3P, and (**c**) TEA aqueous solutions on AISI 52100 steel and alumina ceramic surfaces.

The formation of EOF is caused by the action of the axial electric field on counterions in the EDL. Hence the amplitude and polarity of the zeta potential can considerably impact the magnitude and direction of EOF. To further investigate the influences of additives and their concentrations on capillary electroosmosis, PEG400, MOA-3P, and TEA were selected to test in order to calculate their streaming potential and zeta potential. The streaming potentials of the three additive solutions on the surface of AISI 52100 steel and alumina ceramic are all negative, as shown in Figure 4, indicating that the surface charges of AISI 52100 steel and alumina ceramic in contact with these solutions are negative.

**Figure 5:**
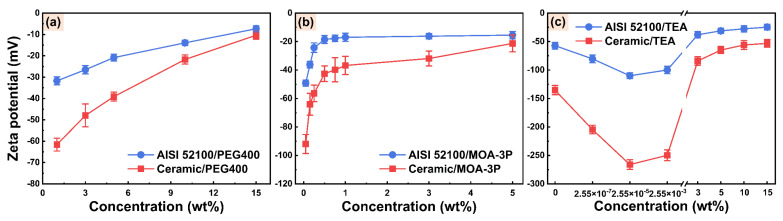
Zeta potentials of (**a**) PEG400, (**b**) MOA-3P, and (**c**) TEA aqueous solutions on AISI 52100 steel and alumina ceramic surfaces.

The total resistance *R_t_* of AISI 52100 steel microchannels filled with these solutions and the EDL conductivities *λ_EDL_* of alumina ceramic microchannels filled with these solutions are listed in Table S1. The zeta potentials of three additive solutions on the surface of AISI 52100 steel and alumina ceramic were then calculated by Equations (2) and (3), respectively. It can be seen from Figure 5 that the zeta potentials of the PEG400, MOA-3P, and TEA solutions on the surfaces of the AISI 52100 steel and alumina ceramic have the same polarity as the streaming potentials. Additionally, in three additive solution systems, the zeta potentials of the AISI 52100 steel and alumina ceramic surfaces show the same trend with concentration, indicating that both materials have similar EDL characteristics in these solutions. However, the absolute values of the zeta potentials of alumina ceramic in these solutions are higher than those for AISI 52100 steel, indicating the charging capacity of alumina ceramic is better than that of AISI 52100 steel.

The relationship between the zeta potential and the concentration of the PEG400 solution is depicted in Figure 5a. The absolute value of the zeta potential decreases as the concentration increases. This is mainly because more polymers are adsorbed to the solid surface with the increasing concentration of PEG400, resulting in the shear plane moving away from the solid surface and the absolute value of the zeta potential decreasing [54,55,56,57]. Similarly, the absolute value of the zeta potential in the MOA-3P solution system decreases with increasing concentration, as shown in Figure 5b. This is because the increase in ion concentration leads to a compression of EDL at the friction material/solution interface, reducing the absolute value of the zeta potential [19,20,58]. Additionally, it can be seen from Figure 5c that with the increasing concentration of TEA solution system, the absolute value of the zeta potential shows a trend of first rising and then decreasing. This is because the rise of TEA concentration increases the pH value, which promotes the adsorption of ions or the dissociation of solid surface groups [59,60]. Consequently, the surface charge density of friction material increases, resulting in an improvement in the absolute value of the zeta potential. However, TEA is a weak base with a low dissociation constant. When the concentration exceeds 3 wt%, the increase of pH with increased concentration is no longer obvious. The EDL becomes thinner with increased concentration, resulting in a decrease in the absolute value of zeta potential of TEA solution in the concentration range of 3–15 wt%. Furthermore, the relationship curves between zeta potential at friction material/solution interface and additive concentration have the same trends as the relationship curves between EOF velocity in alumina ceramic capillary (Figure 3) and additive concentration. This is precisely because the amplitude of zeta potential determines the magnitude of EOF velocity.

### 3.2. Electroosmotic Characteristics of Compound Cutting Fluids

Based on the investigation of the EOF velocities and zeta potentials of PEG400, MOA-3P, and TEA solutions, it is clear that the electroosmotic performance levels of the three additive solutions are quite different. The EOF velocity of TEA is higher than that of deionized water, while the EOF velocity of PEG400 is lower than that of deionized water. Additionally, the EOF velocity of MOA-3P is higher than that of deionized water at low ion concentrations and lower at high ion concentrations. PEG400 and TEA concentrations in the water-based cutting fluid were set at 3 wt% and 3 wt%, respectively, in consideration of the electroosmotic performance of the additives and their content level in the general formula [33,61]. Then, the MOA-3P in the range of 0.05–1 wt% was compounded with 3 wt% PEG400 and 3 wt% TEA to study the electroosmotic performance of compound cutting fluid.

**Figure 6:**
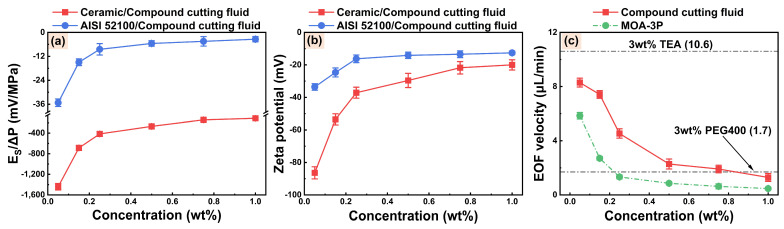
(**a**) Streaming potentials, (**b**) zeta potentials, and (**c**) EOF velocities of compound cutting fluids with different concentrations of MOA-3P.

Figure 6 depicts the streaming potentials, zeta potentials, and EOF velocities of compound cutting fluids with different concentrations of MOA-3P. It can be observed from Figure 6a that the absolute values of streaming potentials on both AISI 52100 steel and alumina ceramic surfaces reduce as the concentrations of MOA-3P increase. According to the total resistance *R_t_* and the EDL conductivities *λ_EDL_* (Table S2), the zeta potentials of AISI 52100 steel and alumina ceramic surfaces in various compound cutting fluids were calculated. It can be observed from Figure 6b that the absolute values of the zeta potential of compound cutting fluid decrease continuously as the MOA-3P concentrations increase from 0.05 to 1 wt%. This is because the EDL is compressed as a result of the rise in ion concentration, which lowers the absolute value of the zeta potential, further suppressing the EOF velocity of the compound cutting fluid, as shown in Figure 6c. Additionally, the increase of MOA-3P concentration can slightly reduce the pH value of a compound cutting-fluid system and increase its dynamic viscosity and bulk conductivity (Figure S3). These changes in physical properties will also reduce the EOF velocity of a compound cutting fluid. Figure 6c also shows that the EOF velocity of compound cutting fluid is greater than that of MOA-3P solution and 3 wt% PEG400 solution, and smaller than that of a 3 wt% TEA solution with better electroosmotic performance. This indicates that the electroosmotic performance of a cutting-fluid system after the compounding of additives with different electroosmotic performance levels can be complementary. To ensure the considerable electroosmotic performance of the compound cutting fluid and taking into account the amount of general EP additive, the concentration of MOA-3P was set at 0.75 wt% in preparing the water-based cutting fluid (named WCF).

To further improve the electroosmotic performance, electroosmotic regulators are often added to the cutting fluid [16,27]. The electroosmotic regulation characteristic of cutting fluid with more complex components is still unclear, which limits the application of the electroosmosis effect in tribology. Therefore, 0–0.8 mmol/L CHAPS or CTAB were added to WCF to study the effects of electroosmotic regulators on the electroosmotic performance and tribological performance of cutting fluid.

### 3.3. Electroosmotic Regulation Characteristics of WCF Cutting Fluids

**Figure 7:**
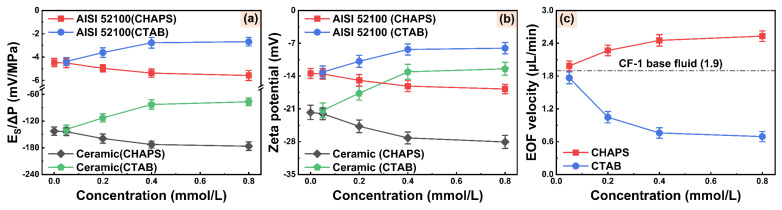
(**a**) Streaming potentials, (**b**) zeta potentials, and (**c**) EOF velocities of WCF cutting fluids with different CHAPS and CTAB concentrations.

Figure 7a,b show the changes in the streaming potentials and zeta potentials of WCF cutting fluid with different electroosmotic regulators. The absolute values of the streaming potential and zeta potential on both AISI 52100 steel/cutting fluid and alumina ceramic/cutting-fluid interfaces gradually increase when CHAPS concentration rises. In contrast, the absolute values of the streaming potential and zeta potential gradually decrease when CTAB concentration rises. Figure 7c depicts the changes in the EOF velocities of WCF cutting fluid with different concentrations of electroosmotic regulators. The EOF velocity of cutting fluid gradually increases with increased CHAPS concentration and tends to be stable at CHAPS concentrations higher than 0.4 mmol/L. The EOF velocity of WCF cutting fluid containing 0.8 mmol/L CHAPS increases by 32.13% compared to the base fluid. In contrast, the addition of CTAB results in a decrease in the EOF velocity. The EOF velocity of WCF cutting fluid containing 0.8 mmol/L CTAB decreases by 63.81% compared to the base fluid. This indicates that the electroosmotic regulators CHAPS and CTAB have significant effects on the EOF velocity of WCF cutting fluid. Based on the trend of zeta potential and EOF, it is clear that the electroosmotic regulators can effectively adjust the electroosmotic characteristics of WCF cutting fluid.

### 3.4. Tribological Performance Characteristics of WCF Cutting Fluids

#### 3.4.1. Effects of Electroosmotic Regulators on the Tribological Performance of WCF Cutting Fluid

**Figure 8:**
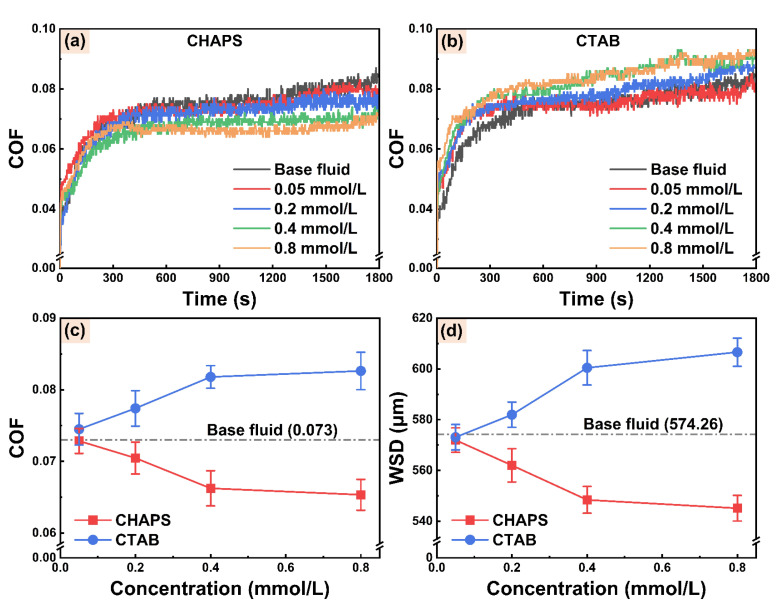
The COF curves for steel–steel friction interface under the lubrication of WCF cutting fluid with different concentrations of (**a**) CHAPS and (**b**) CTAB. The average (**c**) COF and (**d**) WSD for steel–steel friction interface under the lubrication of WCF cutting fluid containing different concentrations of electroosmotic regulators.

Figure 8a,b show the COF curves of the steel–steel friction pair with WCF cutting fluids containing different concentrations of electroosmotic regulators. The COF curves of WCF cutting fluids containing different concentrations of CHAPS and CTAB exhibit a consistent trend, and the COF curve of WCF cutting fluid containing CHAPS is lower than that of containing CTAB. The changes in the average COF of WCF cutting fluid with different concentrations of electroosmotic regulators are depicted in Figure 8c. With the increase of the electroosmotic regulators’ concentration from 0 to 0.8 mmol/L, the average COF of WCF cutting fluid containing CHAPS gradually decreases, reduced by 10.97% compared with the base fluid. Conversely, the average COF of WCF cutting fluid containing CTAB gradually rises, increasing by 12.59% compared with the base fluid. Figure 8d presents the effects of variance in CHAPS and CTAB concentrations on the average WSD of steel–steel friction interface. It can be observed that the changes in the average WSD and COF with the CHAPS or CTAB concentrations have the same trend. As the concentrations of CHAPS and CTAB reach 0.8 mmol/L, the changes in average WSD compared to the base fluid are −5.08% and 5.63%, respectively. The above changes in the tribological performance levels of cutting fluid may be related to the promotion or suppression of the electroosmotic performance of cutting fluid by electroosmotic regulators. The cutting fluid with excellent electroosmotic performance has good permeability at the friction interface, thus showing superior anti-friction and anti-wear effects.

#### 3.4.2. Worn Surfaces Analysis

**Figure 9:**
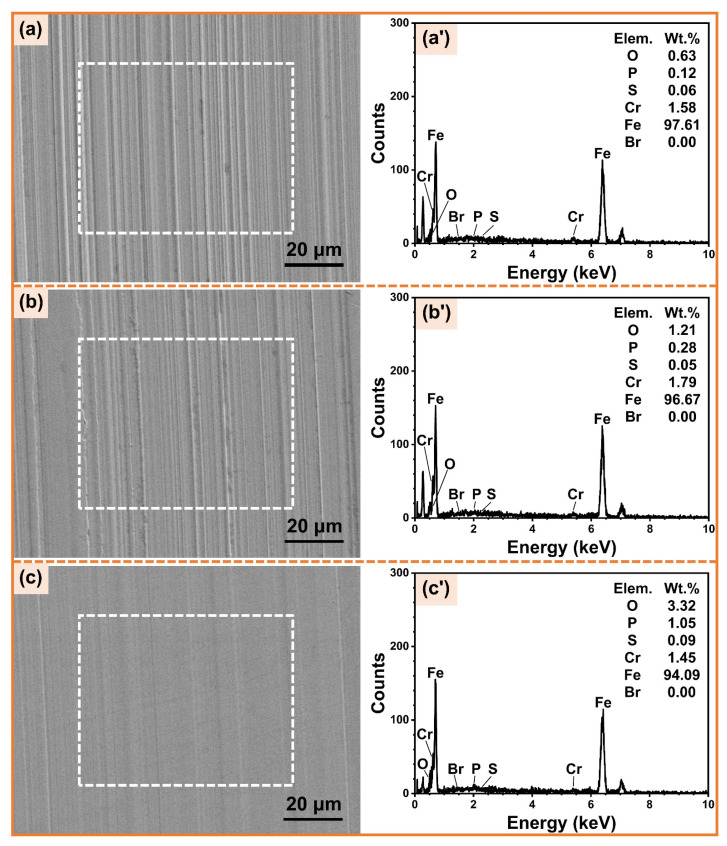
SEM images of worn surfaces and EDS spectra of the dashed areas under the lubrication of (**a**,**a’**) WCF containing 0.8 mmol/L CTAB, (**b**,**b’**) WCF base fluid, and (**c**,**c’**) WCF containing 0.8 mmol/L CHAPS.

Figure 9 depicts the SEM images and EDS spectra of worn surfaces under the lubrication of WCF cutting fluid alone and WCF cutting fluid containing 0.8 mmol/L electroosmotic regulators. It can be observed that furrows appear on the worn surfaces due to abrasive wear. The worn surface lubricated by the WCF cutting fluid containing CTAB presents more severe abrasive wear and deeper furrows, while the worn surface lubricated by the WCF cutting fluid containing CHAPS shows smoother and shallower furrows. The results further indicate the superior anti-wear effect of WCF cutting fluid containing CHAPS. According to the EDS energy spectra measured in the dashed areas, only 0.05–0.09 wt% of S and 0 wt% of Br are present on the worn surfaces. CHAPS contains S, while CTAB contains Br, indicating that only a very small amount of electroosmotic regulators are involved in the lubrication process. The maximum mass fractions of CHAPS and CTAB in cutting fluid are only 0.0492 wt% and 0.0292 wt%, respectively. Therefore, the anti-friction and anti-wear performance levels of cutting fluid are largely not affected by the lubrication performance of the electroosmotic regulators themselves. The improvement in the tribological performance of WCF cutting fluid may be mainly due to more cutting fluid penetrating into the steel–steel friction interface. Additionally, the content of the elements O and P on the worn surface when using the cutting fluid containing CHAPS is significantly higher than those for the other two cases. PEG400, MOA-3P, and TEA in WCF cutting fluid all contain O, and MOA-3P contains P, indicating that the addition of CHAPS improves the electroosmotic performance of cutting fluid at the friction interface. More O and P are thereby supplied to participate in the anti-wear process and form a lubricating film on the worn surface [16,62]. However, the content of O and P on the worn surface using the cutting fluid containing CTAB decreases, indicating that the addition of CTAB suppresses the electroosmotic performance of cutting fluid at the friction interface, which further inhibits the formation of lubricating film and results in worse tribological performance.

### 3.5. Electroosmotic Regulation Mechanism

**Figure 10:**
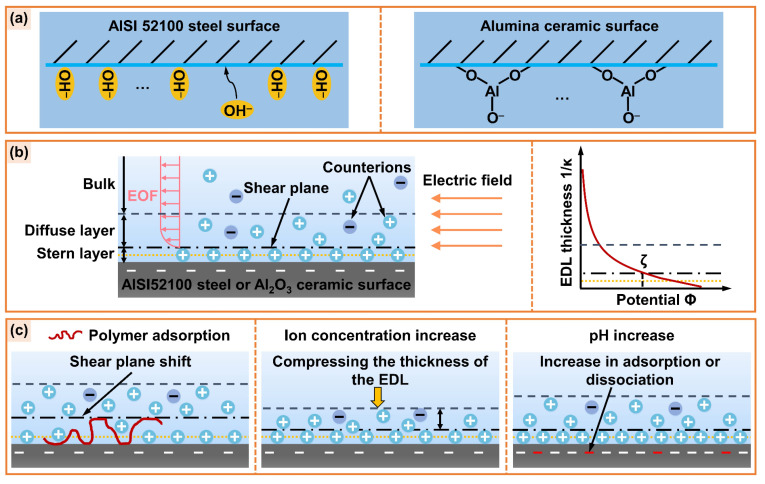
Schematic diagram of (**a**) the surface charge of AISI 52100 steel and alumina ceramic, (**b**) the EDL and its potential distribution, (**c**) the effects of additives on the zeta potential of the EDL.

Figure 10a illustrates the mechanism of the surface charge of AISI 52100 steel and alumina ceramic. The contact of both materials with cutting fluid leads to the excess negative charge on the solid surface due to the adsorption of OH^−^ ion or dissociation of surface Al-OH groups [63,64]. The negatively charged surface will cause the rearrangement of counterions in the liquid phase, forming an EDL structure at the solid/liquid interface. Under the action of the applied electric field, the cations in the diffusion layer will migrate to the negative electrode, together with the surrounding solvent molecules, through the viscous resistance to form EOF, as presented in Figure 10b. The zeta potential in the EDL is a crucial factor affecting EOF. The zeta potential on the solid surface is mainly affected by the composition of the solution system, the ion concentration, and the pH value. Figure 10c depicts the influence of additives on the electroosmotic performance. After the polymer is adsorbed to the solid surface, the steric hindrance effect is generated. Then, the shear plane moves away from the solid surface, and the absolute value of zeta potential decreases, thereby suppressing EOF [55,56]. The increase of ion concentration in the solution system will lead to a compression of EDL, and the counterions in the diffusion layer penetrate the Stern layer under the action of electrostatic repulsion. As a result, the charge density in the diffusion layer will decrease, lowering the absolute value of zeta potential and EOF [19,60]. Additionally, the rise of the pH value in the solution system can promote the specific adsorption of OH^−^ ions on the AISI 52100 steel surface or the dissociation of Al-OH groups on the alumina ceramic surface, which increases the surface charge density and the absolute value of zeta potential, thereby promoting EOF [59,60].

**Figure 11:**
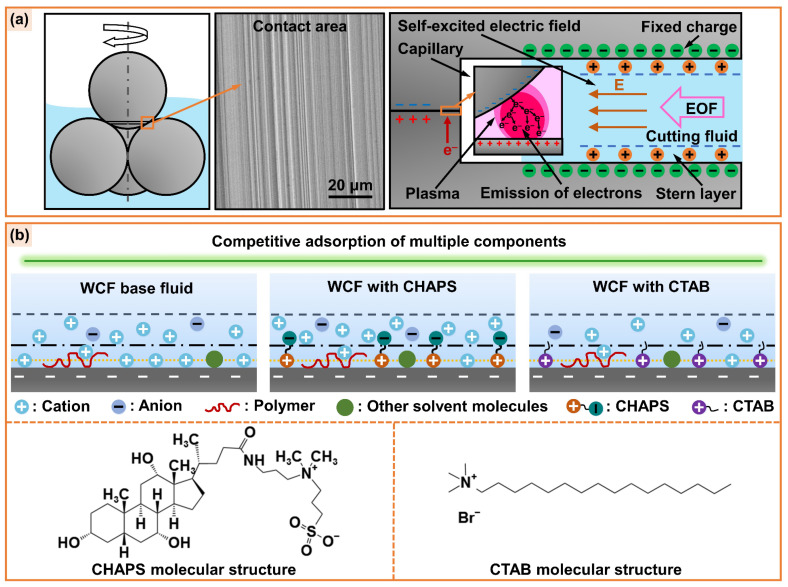
Schematic diagram of (**a**) capillary electroosmosis of cutting fluid at friction interface and (**b**) the regulation mechanism of electroosmotic regulators CHAPS and CTAB.

Figure 11a depicts the mechanism of capillary electroosmosis of cutting fluid at the friction interface. Numerous capillaries are formed at the friction interface during the friction process as a result of the scratching and ploughing action of the upper and lower friction pairs. The cutting fluid penetrates into the friction interface through traditional capillary penetration, thus forming an EDL at the capillary wall/cutting-fluid interface. In addition, the triboelectric phenomenon can generate an axial self-excited electric field greater than 150 V/cm in the interfacial capillary [14], which in turn induces capillary electroosmosis of the cutting fluid. Figure 11b presents the electroosmotic regulation mechanism of cutting fluid. The electroosmotic regulators CHAPS and CTAB are zwitterionic and cationic surfactants, respectively. Their cationic groups are adsorbed to the capillary wall by electrostatic interaction, which can change the EDL structure at the capillary wall/cutting-fluid interface. The anionic groups of CHAPS accumulate near the capillary wall and attract cations in solution, which increases the charge density in the diffusion layer, thus increasing the absolute value of the zeta potential and promoting EOF [15,25]. Hence, the addition of CHAPS can improve the penetration ability of cutting fluid at the friction interface to a certain extent, showing superior anti-friction and anti-wear performance. The hydrophobic carbon chain of CTAB neutralizes the wall charge, which reduces the absolute value of zeta potential and suppresses EOF [24,25]. Therefore, the addition of CTAB suppresses the penetration of cutting fluid at the friction interface, which is associated with poor anti-friction and anti-wear performance. The water-based cutting fluid (taking WCF cutting fluid as an example) contains various additives and many organic ions. These ions can be adsorbed to the charged surface by electrostatic or non-electrostatic action, forming a competitive relationship with electroosmosis regulators and affecting their regulation effect on electroosmosis [65].

## 4. Conclusions

In this paper, the EOF velocities and zeta-potential characteristics of water-based cutting-fluid additives and their compound cutting fluids were investigated. And the mechanisms of additives and compound cutting fluids for electroosmotic performance were revealed, which provides a reference for the formulation design of cutting fluids based on the electroosmosis effect. In addition, the effects of electroosmotic regulators on the tribological performance of cutting fluid were verified. The main conclusions are as follows:

(1) MOA-3P and TEA are used as EP additive and pH buffer, respectively, for water-based cutting fluid. Their excessive ion concentration will lead to a compression of EDL at the friction material/solution interface, causing the absolute value of the zeta potential and EOF to decrease.
(2) PEG400 is used as a lubricity additive for water-based cutting fluid. Its chain segments are adsorbed to the surface of friction material to produce steric hindrance, which makes the shear plane move away from the solid surface, and the absolute value of zeta potential and EOF decrease.
(3) The specific adsorption of OH^−^ ions on the AISI 52100 steel surface and the dissociation of Al-OH groups on the alumina ceramic surface can be promoted to some extent by an increase in pH value in a water-based cutting-fluid system, which increases the absolute value of zeta potential and EOF.
(4) The electroosmotic performance of water-based cutting fluid can be further adjusted by adding electroosmotic regulators. The electroosmotic promoter CHAPS increases the absolute value of zeta potential by electrostatic adsorption, which promotes the penetration ability of cutting fluid in the capillary zone of the friction interface, improving the tribological performance of cutting fluid at the steel–steel interface. The electroosmotic suppressant CTAB reduces the absolute value of the zeta potential and suppresses EOF, exhibiting poor tribological performance.

## Figures and Tables

**Table 1:** Information on major chemical reagents.

Types of Additives	Reagents	Chemical Formulas	Manufacturers
Lubricity additive	Polyethylene glycol (PEG200, PEG300, PEG400, PEG600 and PEG800)	HO(CH_2_CH_2_O)_n_H	Shanghai Macklin Biochemical Co., Ltd., Shanghai, China
EP additive	Potassium tetraborate (B_4_K_2_O_7_·4H_2_O)	H_8_B_4_K_2_O_11_	Sinopharm Chemical Reagent Co., Ltd., Shanghai, China
	Fatty alcohol ether phosphate (MOA-3P and MOA-9P)	RO(CH_2_CH_2_O)_n_PO(OH)_2_ and [RO(CH_2_CH_2_O)_n_]_2_PO(OH)	Jiangsu Haian Petrochemical Plant, Nantong, China
pH buffer	Triethanolamine (TEA)	C_6_H_15_NO_3_	Shanghai Aladdin Biochemical Technology Co., Ltd., Shanghai, China
Electroosmotic promoter	3-[(3-cholamidopropyl)-dimethylammonio]-1-propanesulfonate (CHAPS)	C_32_H_58_N_2_SO_7_	Shanghai Macklin Biochemical Co., Ltd., Shanghai, China
Electroosmotic suppressant	Cetyltrimethylammonium bromide (CTAB)	C_19_H_42_BrN	Shanghai Macklin Biochemical Co., Ltd., Shanghai, China

**Table 2:** The tribological test conditions.

Item	Value
Material	AISI 52100 steel ball (diameter 12.7 mm, hardness 59–61 HRC)
Load	147 N
Rotational speed	1200 rpm
Time	30 min
Types of cutting fluids	WCF base fluid
	WCF base fluid + x mmol/L CHAPS (x = 0.05, 0.2, 0.4, 0.8)
	WCF base fluid + x mmol/L CTAB (x = 0.05, 0.2, 0.4, 0.8)

## Data Availability

Data will be made available on request.

## Supplementary Materials

The following supporting information can be downloaded at: https://www.mdpi.com/article/10.3390/mi14112029/s1, Figure S1: Dynamic viscosities of (a) lubricity additive, (b) EP additive, and (c) pH buffer aqueous solutions; Figure S2. Bulk conductivities of (a) lubricity additive, (b) EP additive, and (c) pH buffer aqueous solutions; Figure S3. (a) Dynamic viscosities, (b) bulk conductivities, and (c) pH values of compound cutting fluid with different concentrations of MOA-3P; Table S1: Total resistance *R_t_* of AISI 52100 steel microchannel and the EDL conductivity *λ_EDL_* of alumina ceramic microchannel when filled with PEG400, MOA-3P, and TEA solutions; Table S2 Total resistance *R_t_* of AISI 52100 steel microchannel and EDL conductivity *λ_EDL_* of alumina ceramic microchannel when filled with compound cutting fluid; Table S3 Total resistance *R_t_* of AISI 52100 steel microchannel and EDL conductivity *λ_EDL_* of alumina ceramic microchannel when filled with WCF cutting fluid.
