# Supplementary material for: Effects of the Electric Double Layer Characteristic and Electroosmotic Regulation on the Tribological Performance of Water-Based Cutting Fluids
Source: Micromachines (Basel). 2023 Oct 31;14(11):2029. doi: 10.3390/mi14112029 (PMC10673424; doi:10.3390/mi14112029)
Supplement: Supplementary file 1 [file micromachines-14-02029-s001.zip › micromachines-2681964-supplementary.pdf]

Supplementary materials

1. Total resistance R_t of AISI 52100 steel microchannel and EDL conductivity λ_{EDL} of alumina ceramic microchannel when filled with solution

Table S1 Total resistance R_t of AISI 52100 steel microchannel and the EDL conductivity λ_{EDL} of alumina ceramic microchannel when filled with PEG400, MOA-3P, and TEA solutions

Types of additives	Parameters	Value							
PEG400	Concentration (wt%)	1	3	5	10	15	-	-	-
	R_t (kΩ)	176	174	180	184	179	-	-	-
	λ_{EDL} (μS/cm)	49	49.8	50.9	50	52.1	-	-	-
MOA-3P	Concentration (wt%)	0.05	0.15	0.25	0.5	0.75	1	3	5
	R_t (kΩ)	115	68	61	49	43	35	23	19
	λ_{EDL} (μS/cm)	280.9	336.4	385	494	640	746	1130	1198
TEA	Concentration (wt%)	0	2.55×10^{-7}	2.55×10^{-5}	2.55×10^{-3}	3	5	10	15
	R_t (kΩ)	175	148	124	105	74	67	55	47
	λ_{EDL} (μS/cm)	110.7	132	171.3	176.8	182.7	191.3	195.7	199.3

Table S2 Total resistance R_t of AISI 52100 steel microchannel and EDL conductivity λ_{EDL} of alumina ceramic microchannel when filled with compound cutting fluid

Parameters	Value					
Concentration of MOA-3P (wt%)	0.05	0.15	0.25	0.5	0.75	1
R_t (kΩ)	110	65	57	45	40	33
λ_{EDL} (μS/cm)	303.9	385.6	432	508	673	768

Table S3 Total resistance R_t of AISI 52100 steel microchannel and EDL conductivity λ_{EDL} of alumina ceramic microchannel when filled with WCF cutting fluid

Types of additives	Parameters	Value				
CHAPS	Concentration (mmol/L)	0	0.05	0.2	0.4	0.8
	R_t (kΩ)	40	40.1	40	39.8	39.9
	λ_{EDL} (μS/cm)	673	676	685	695.3	701.6
CTAB	Concentration (mmol/L)	0	0.05	0.2	0.4	0.8
	R_t (kΩ)	40	39.9	40.1	39.9	39.7
	λ_{EDL} (μS/cm)	673	679	689.6	697.6	716

2. Physicochemical properties of additive aqueous solutions

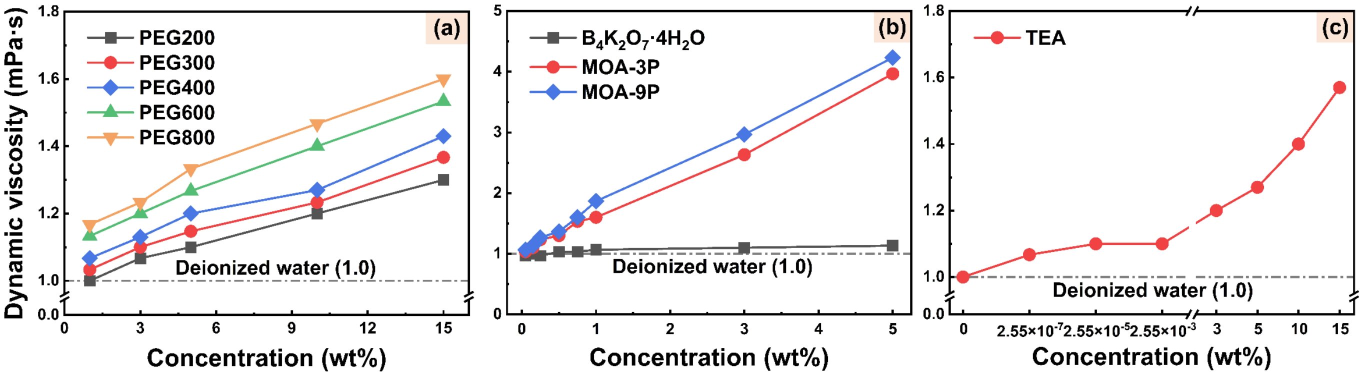

Figure S1. Dynamic viscosities of (a) lubricity additive, (b) EP additive, and (c) pH buffer aqueous solutions.

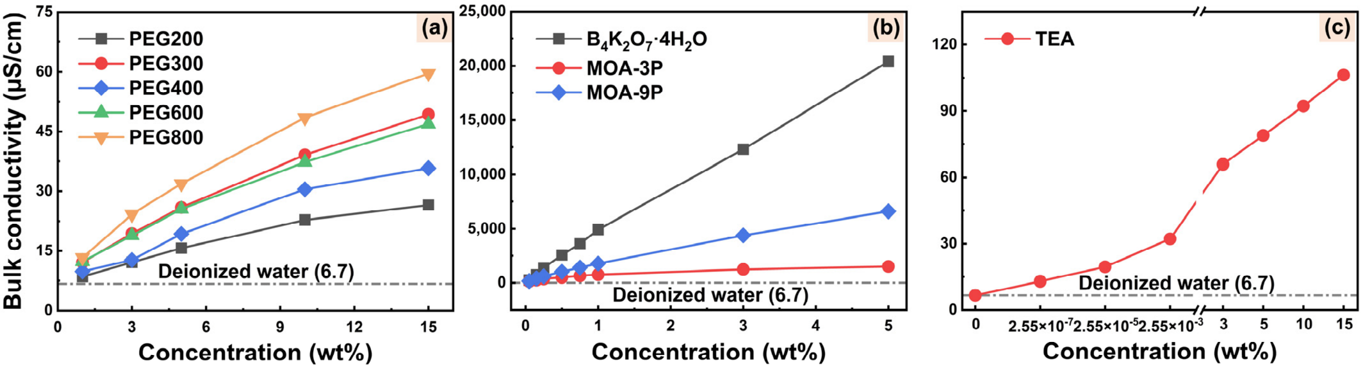

Figure S2. Bulk conductivities of (a) lubricity additive, (b) EP additive, and (c) pH buffer aqueous solutions.

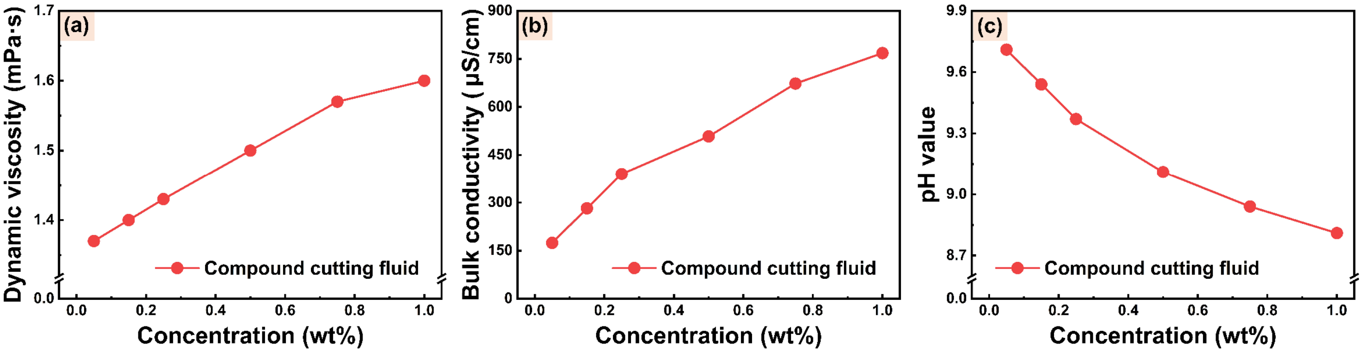

Figure S3. a) Dynamic viscosities, (b) bulk conductivities, and (c) pH values of compound cutting fluid with different concentrations of MOA-3P.
